# Functional Contribution of the Medial Prefrontal Circuitry in Major Depressive Disorder and Stress-Induced Depressive-Like Behaviors

**DOI:** 10.3389/fnbeh.2021.699592

**Published:** 2021-06-21

**Authors:** Thibault P. Bittar, Benoit Labonté

**Affiliations:** ^1^CERVO Brain Research Centre, Québec, QC, Canada; ^2^Department of Psychiatry and Neuroscience, Faculty of Medicine, Université Laval, Québec, QC, Canada

**Keywords:** major depressive disorder, functional studies, neuronal circuit, chronic stress (or “stress”), mouse model, sex differences

## Abstract

Despite decades of research on the neurobiology of major depressive disorder (MDD), the mechanisms underlying its expression remain unknown. The medial prefrontal cortex (mPFC), a hub region involved in emotional processing and stress response elaboration, is highly impacted in MDD patients and animal models of chronic stress. Recent advances showed alterations in the morphology and activity of mPFC neurons along with profound changes in their transcriptional programs. Studies at the circuitry level highlighted the relevance of deciphering the contributions of the distinct prefrontal circuits in the elaboration of adapted and maladapted behavioral responses in the context of chronic stress. Interestingly, MDD presents a sexual dimorphism, a feature recognized in the molecular field but understudied on the circuit level. This review examines the recent literature and summarizes the contribution of the mPFC circuitry in the expression of MDD in males and females along with the morphological and functional alterations that change the activity of these neuronal circuits in human MDD and animal models of depressive-like behaviors.

## Introduction

Recent estimates from the World Health Organization (WHO) revealed that more than 300 million people of all ages suffer from major depressive disorder (MDD) worldwide (World Health Organization, 2018). Despite its enormous economic and human impact, MDD remains a highly pervasive and co-morbid disorder for which the scientific community has globally failed to provide underlying genetic and molecular etiologies (Fava and Kendler, 2000; Kendler et al., 2006; Flint and Kendler, 2014). This is particularly true considering low treatment efficacy despite the number of drugs that have been developed to treat MDD during the last 50 years (Nestler, 1998; Swedish Council on Health Technology Assessment, 2004). This persistent incapacity to treat the disease efficiently results in part from the heterogeneity of the disease and our poor understanding of the molecular and functional mechanisms underlying its expression.

MDD is a highly heterogeneous disorder characterized clinically by the expression of persistent depressed mood, anxiety, anhedonia, cognitive impairments, weight changes, fatigue, feeling of worthlessness, recurrent thoughts of death, and suicidal ideation (American Psychiatric Association, 2013, p. 5). These symptoms vary widely across patients and their expression evolves with the chronicity of the disease. Consequently, previous attempts at clustering patients based on their clinical profiles globally failed to identify more homogeneous groups of patients (Fried and Nesse, 2015). Another level of complexity and heterogeneity comes from the fact that MDD is a sexually dimorphic disease. Recent estimates have evaluated the prevalence of MDD to be 2–3 times higher in women compared to men (Weissman et al., 1993, 1996; Kim et al., 2015; World Health Organization, 2018). Clinical studies report women to exhibit higher scores of depression, younger age of onset, a higher number of depressive episodes, and relapse rates than men (Kessler et al., 1993, 2005; Nazroo et al., 1997; Parker et al., 2014; Breslau et al., 2017). Women have also been shown to exhibit higher rates of comorbid anxiety, cardiovascular disease risks, and atypical symptoms like sadness, sexual function impairment, susceptibility to physical pain, and vegetative state (Kornstein et al., 2000a; Lai, 2011; Shah et al., 2014; Bandelow and Michaelis, 2015; Kim et al., 2015; Karpyak et al., 2016). In contrast, men often exhibit comorbid substance abuse including alcohol and drugs (Grant et al., 2016; Karpyak et al., 2016). Men also display poorer impulse control and increased anger and aggressivity, and are more prone to take risks than women (Rice et al., 2013; Cavanagh et al., 2017; Athanasiadis et al., 2018). Furthermore, this sexual dimorphism is also observed at the treatment level with studies suggesting better responses in men treated with tricyclic antidepressant drugs (TCA), and selective serotonin reuptake inhibitors (SSRI) in women (Kornstein et al., 2000b; Khan et al., 2005; Young et al., 2009; Dalla et al., 2010), although these findings have been contested (Thase et al., 2005; Sramek et al., 2016).

A growing body of evidence now suggests that the expression of different symptoms in males and females may results from the alterations of distinct pathways and circuits in the brain with the medial prefrontal cortex (mPFC) acting as a hub structure. For instance, elevated functional connectivity of the mPFC with the posterior cingulate cortex and angular gyrus is associated with rumination while hypoactivation of the ventral striatum associates with the expression of anhedonia (Williams, 2016). Interestingly, hyperactivity of the ACC has also been associated with better responses to antidepressants in MDD patients (Phillips et al., 2015). Finally, functional studies in treatment-resistant MDD patients have consistently revealed hyperactivity in the mPFC (Hadas et al., 2019; Morris et al., 2020) which can be reversed by the stimulation of the subcallosal cingulate cortex along with a reversal of the depressive symptomatology (Holtzheimer et al., 2017). Interestingly, changes in the activity of these pathways have been consistently associated with morphological (Drevets et al., 2008; Zhao et al., 2014) and sex-specific molecular (Gray et al., 2015; Labonté et al., 2017; Seney et al., 2018) changes that further support the role of the mPFC as a hub region in mediating the expression of MDD in men and women.

The hub status of the mPFC is further strengthened by anatomical and functional studies performed in animal models of chronic stress. The mPFC integrates neuronal inputs from several brain regions (Zhang et al., 2010; Gabbott et al., 2012; Linley et al., 2013) and sends neuronal projections to several cortical and limbic structures (Gabbott et al., 2005; Kim et al., 2017). Through these functional connections, the mPFC controls several cognitive and behavioral functions such as attention, habit formation, decision making, long-term memory, working memory, aversive and appetitive stimuli processing, and emotional and inhibitory control (Bechara et al., 2000; Ridderinkhof et al., 2004; Euston et al., 2012; Kumar et al., 2013; Laubach et al., 2015; Moorman et al., 2015; Wellman and Moench, 2019). Importantly, alterations of these fundamental processes are believed to result in the expression of depressive-like behaviors (Keedwell et al., 2005; Murrough et al., 2011). This has been strongly supported by an increasing number of functional studies in animal models of chronic stress dissecting the contribution of the different neuronal circuits of the mPFC in the expression of depressive-like behaviors in males and females (Vialou et al., 2014; Bagot et al., 2015; Dolzani et al., 2018; Liu W.-Z. et al., 2020; Marcus et al., 2020; Bittar et al., 2021). Here, we review the literature on the involvement of the mPFC and its neuronal circuitry in mediating the expression of depressive-like behaviors. We start by defining the anatomical and cytoarchitectural organization of the mPFC circuitry. We then elaborate on the functional and morphological impact of chronic stress on excitatory pyramidal neurons and GABAergic interneurons in the mPFC. Finally, we provide an overview of the most recent studies supporting the role of mPFC circuits in mediating the expression of specific behavioral phenotypes in animal models of depressive-like behaviors.

## Cell Type-Specific Distribution and Anatomical Organization of the mPFC

### Anatomical Organization

Consistent with its crucial role in higher executive functions, the mPFC is more developed in higher order mammals, and exhibit more elaborate behaviors than rodents (Kerney et al., 2017; Noonan et al., 2018). In humans, the mPFC is composed of different structures from dorsal to ventral: the dorsal anterior cingulate cortex (dACC, Brodmann area 32), the pregenual anterior cingulate cortex (pACC, Brodmann area 24), and the subgenual cingulate cortex (sACC, Brodmann area 25) (aan het Rot et al., 2009). The dACC is involved in detecting and filtering salient stimuli (Menon and Uddin, 2010), while the pACC and sACC are relevant to emotion processing and regulation of attention (Palomero-Gallagher et al., 2019). In contrast, the rodent mPFC is subdivided into four anatomically and functionally distinct sub-regions (Heidbreder and Groenewegen, 2003). The dorsal part, named dorsomedial prefrontal cortex (dmPFC), encompasses the secondary motor area (M2) and the anterior cingulate cortex (ACC). The ventral part, named ventromedial prefrontal cortex (vmPFC), includes the prelimbic cortex (PLC) and the infralimbic cortex (ILC). The evolution of the brain complexity and morphology (curvature notably) across species renders the comparison between humans and rodents difficult although equivalent structures (functionally and anatomically) have been suggested. The rodent PLC is described as the human pACC and the rodent ILC as the human equivalent of the sACC (Bicks et al., 2015; Ko, 2017; Laubach et al., 2018). Therefore, these rodent structures fill the same functions as their human counterparts: attention, habit formation, decision making, long-term memory, working memory, emotional control, and inhibitory control (Bechara et al., 2000; Ridderinkhof et al., 2004; Euston et al., 2012; Kumar et al., 2013; Laubach et al., 2015; Moorman et al., 2015; Wellman and Moench, 2019). Additionally, the mPFC is also thought to exert top-down executive control over aversive and appetitive stimuli processing (Maroun, 2013) driven by limbic systems regulating stress responses (Price and Drevets, 2012; Riga et al., 2014).

### Cellular Heterogeneity

The mPFC is composed of two genetically distinct neuronal populations, consisting of excitatory pyramidal neurons (80–90%) and GABAergic inhibitory interneurons (10-20%) (Nieuwenhuys, 1994; Rudy et al., 2011). Pyramidal neurons in the mPFC produce glutamate as a neurotransmitter and express Ca^2+^/calmodulin kinase II (CaMKII) (Erondu and Kennedy, 1985; Burgin et al., 1990; Jones et al., 1994; Wang et al., 2013). Studies on rodents revealed that glutamatergic pyramidal neurons in the mPFC are derived from progenitors called radial glial cells (RGCs), located in the ventricular zone at early embryonic stages and maturating while migrating to the neocortical area including the mPFC (Meyer et al., 2000; Marín and Müller, 2014).

The remaining 10–20% are composed of different subtypes of inhibitory interneurons expressing gamma-aminobutyric acid (GABA) as a neurotransmitter (Markram et al., 2004; Narayanan and Laubach, 2017). GABA interneurons of the neocortex arise from RGCs located in the medial ganglionic eminence and the preoptic area during early embryonic stages (Zhu et al., 1999; Wonders and Anderson, 2006; Marín and Müller, 2014). GABA interneurons are composed of different subtypes with heterogeneous morphology, distribution, physiological properties, organization, and connectivity (Petilla Interneuron Nomenclature Group, 2008). These interneurons can be divided into one of the following three non-overlapping classes including those co-expressing the calcium-binding protein parvalbumin (PV, 40%), the neuropeptide somatostatin (SST, 30%), or the vasoactive intestinal peptide (VIP, 30%) (Rudy et al., 2011). VIP neurons are also expressing the specific serotonin receptor 5-HT3A (Rudy et al., 2011) and some PV neurons also express calretinin (Caballero et al., 2014). Interneuron subtypes follow an intracortical distribution and play different roles. VIP and a subset of SST interneurons are found in superficial layers I and II/III. Their role is to modulate excitatory inputs on the dendrites. They mediate lateral inhibition and regulate mPFC long-range inputs by contacting the dendritic tufts. PV interneurons located in deeper layers V and VI regulate the pyramidal neurons’ outputs by contacting the perisomatic regions of the pyramidal neurons. They control the firing synchronization and spike timing of the excitatory neurons in the area. A distinct population of SST interneurons in layers V and VI regulates the activity of these PV interneurons, contributing to the regulation of neuronal outputs. Finally, another population of SST neurons, also located in layers V and VI, assures negative feedback from the axon to the dendrites of pyramidal neurons (Murayama et al., 2009; Larkum, 2013). See the following review for more detailed information (Fee et al., 2017).

### Laminar Organization

**FIGURE 1:**
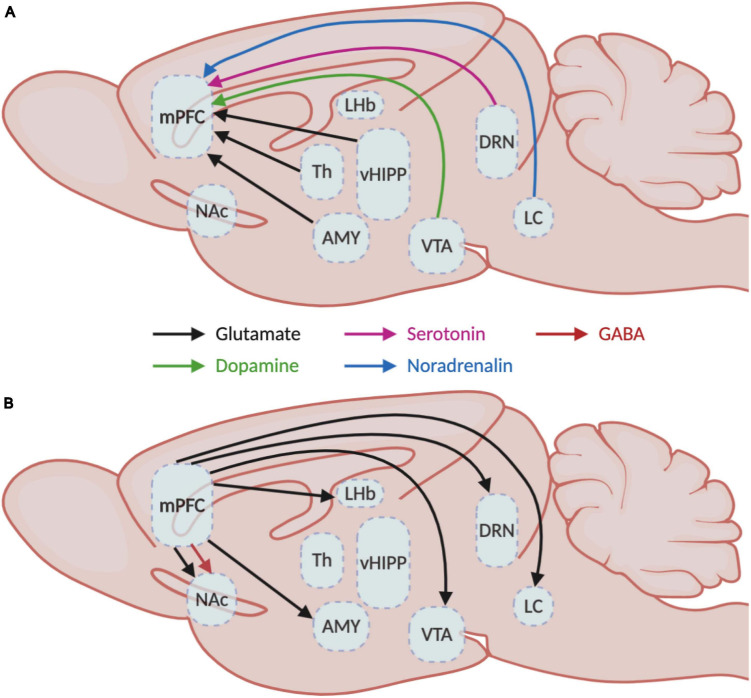
Medial prefrontal cortex inputs and outputs. Schematic representation of the **(A)** afferent and **(B)** efferent projections in the medial prefrontal cortex with their respective neurotransmitter systems. AMY, amygdala; DRN, dorsal raphe nucleus; LC, locus coeruleus; LHb, lateral habenula; mPFC, medial prefrontal cortex; NAc, nucleus accumbens; Th, thalamus; vHIPP, ventral hippocampus; VTA, ventral tegmental area.

**FIGURE 2:**
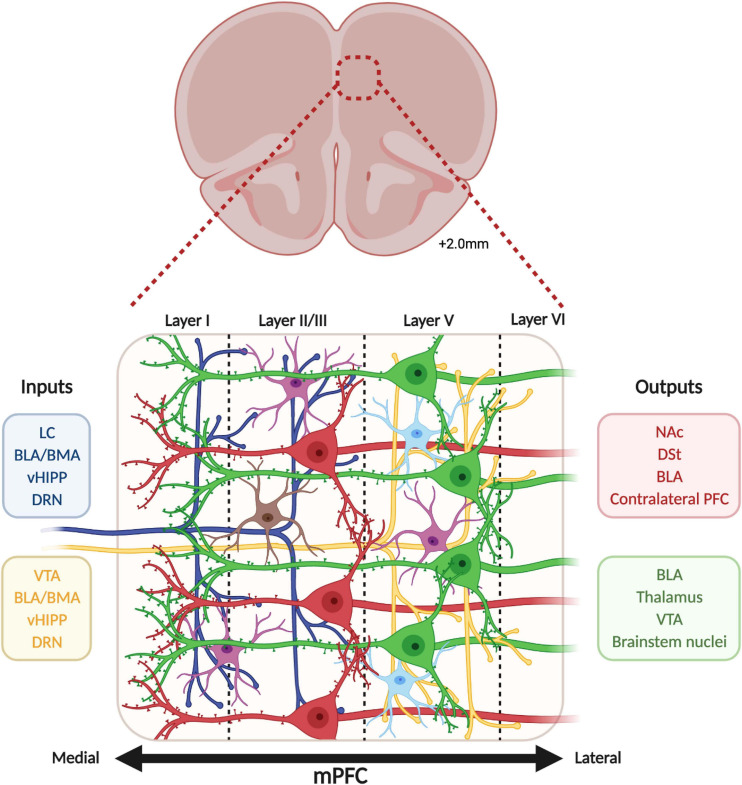
Cellular organization of the medial prefrontal cortex. The medial prefrontal cortex is organized in layers, numbered I (medial, close to the midline) to VI (lateral). Pyramidal glutamate neurons are lying in layers II/III (red), V (green), and VI (not represented), while GABAergic interneurons (somatostatin in purple, parvalbumin in blue, and vasoactive intestinal peptide in brown) are spread in layers I, II/III and V. The mPFC receives layer-specific inputs (dark blue and yellow) from locus coeruleus (LC), basolateral and basomedial amygdala (BLA and BMA), ventral hippocampus (vHIPP), dorsal raphe (DRN) and ventral tegmental area (VTA) while mPFC pyramidal neurons send projections (red and green) to the nucleus accumbens (NAc), dorsal striatum (DSt), BLA, contralateral PFC, thalamus, VTA and brainstem nuclei.

The human mPFC is organized in six layers while rodents lack layer IV (Uylings et al., 2003). Pyramidal neurons are found in layers II/III, IV, V, and VI while GABAergic interneurons populate layers II/III and V predominantly (Douglas and Martin, 2004). Even though the mPFC mainly receives afferences in superficial layers I and II/III, the deep layers V and VI also receive long-range cortical and sub-cortical inputs (Douglas and Martin, 2004; Hoover and Vertes, 2007). These afferences communicate with mPFC neurons through different neurotransmitter systems such as glutamatergic projections from the hippocampus, amygdala, and thalamus (Goldman-Rakic et al., 1984; Barbas and Olmos, 1990; Gabbott et al., 2012), dopaminergic projections from the ventral tegmental area (VTA) (Christie et al., 1985), serotonergic projections from the dorsal raphe nuclei (DRN) (Van Bockstaele et al., 1993) and noradrenergic projections from the locus coeruleus (LC) (Runnels et al., 2009; Figure 1A). These afferences follow a precise topological organization. For instance, inputs from the LC concentrate mainly in layers I and II/III, dopaminergic nerve terminals from the VTA populate preferentially layers V and VI (Vander Weele et al., 2018; Quessy et al., 2021), the amygdala innervates mainly layers II and V (Aggleton et al., 2015; McGarry and Carter, 2016), serotonergic inputs arrive mainly in layers I and V/VI and sparser proportions in layer II/III (Linley et al., 2013), and projections from the hippocampus are located in layers II/III and V (Liu and Carter, 2018; Figure 2).

In return, mPFC neurons send glutamatergic projections to nucleus accumbens (NAc), hippocampus, amygdala, lateral habenula, thalamus and hypothalamus, DRN, VTA, periaqueducal gray (PAG), and LC (Porrino and Goldman-Rakic, 1982; Arnsten and Goldman-Rakic, 1984; Takagishi and Chiba, 1991; Gabbott et al., 2005; Gonçalves et al., 2009; Vander Weele et al., 2018) and also GABAergic projections to the NAc (Lee et al., 2014; Figure 1B). Interestingly, a clear dichotomy in the laminar organization of these projections can be observed between cortico-cortical and cortico-fugal projecting pyramidal neurons (Marín and Müller, 2014). Cortico-cortical neurons from layers II/III project to ipsilateral and contralateral cortical areas, while cortico-fugal neurons spread across layers II/III, V, and VI project mainly to subcortical regions. Subsequent differences can be made between cortico-fugal neurons (Figure 2) with NAc-projecting mPFC pyramidal neurons being mainly located in layers II/III and VTA-projecting mPFC neurons being mainly enriched in sublayers V (Gabbott et al., 2005; Kim et al., 2017; Bittar et al., 2021).

Together, the cellular diversity of the mPFC following a complex laminar and anatomical distribution is ideally organized to integrate the diverse sensory and emotional information coming from other brain regions and redirect it to other structures to drive behavioral responses to environmental challenges. In the next section of this review, we will elaborate on the morphological and functional consequences of chronic stress that have been described in the mPFC of males and females.

## The Impact of Chronic Stress on mPFC Neuronal Network’s Integrity

### Morphological Alterations

Previous studies performed on post-mortem tissue from MDD patients reported several neuroanatomical alterations in the mPFC. For instance, gray matter is reduced in the mPFC of patients suffering from MDD across its different sub-structures (Zhao et al., 2014; Kandilarova et al., 2019). Accordingly, morphological studies in human MDD post-mortem tissue revealed a consistent decrease in the number and length of dendrites and lower spine density in mPFC pyramidal neurons of depressed patients (Drevets et al., 2008; Kang et al., 2012). Although these alterations have been reported mainly in the male brain, most of the studies did not differentiate men from women (Rajkowska et al., 1999) and it is, therefore, difficult to conclude if these variations are sex-specific or not.

These findings in humans have been supported by studies in animal models of chronic stress. For instance, male rats exposed to chronic restraint stress exhibit a 20% reduction of the apical dendritic length and branch number (Cook and Wellman, 2004; Radley et al., 2004; Brown et al., 2005; Hains et al., 2009) with a significant reduction in spine density, volume and surface area in pyramidal neurons from layer II/III of the PLC and ACC (Radley et al., 2008; Hains et al., 2009; Duman and Duman, 2015). Interestingly, a similar decrease in spine density has been observed in susceptible but not resilient male mice following chronic social defeat stress (CSDS) (Qu et al., 2018) and in both males and females following prolonged social isolation (Sarkar and Kabbaj, 2016) in PLC pyramidal neurons but not in ILC. Noticeably, the acute administration of ketamine was shown to rescue spine density deficits in isolated males, but not females, and to reverse anhedonic behaviors and behavioral despair in both sexes (Sarkar and Kabbaj, 2016). These results suggest that the therapeutic action of this fast-acting antidepressant may be mediated through different molecular and functional mechanisms in males and females. Additionally, as it was shown in the human brain, these morphological changes have been associated with a decrease of cytoarchitectural gene expression and protein levels such as TUBB4 (Kang et al., 2012) in men and women.

More recently, exposure to 21 days of chronic variable stress (CVS) has been shown to induce sex-specific modifications in the dendritic complexity of two distinct pyramidal populations in the mPFC, namely NAc- or VTA-projecting mPFC neurons (Bittar et al., 2021). Indeed, in females, CVS was shown to decrease dendritic arborization much more importantly in NAc-projecting mPFC while VTA-projecting neurons showed a greater dendritic retraction in stressed males than females (Bittar et al., 2021). Together, this supports the idea that stress interferes with the morpho-functional properties of specific neuronal pathways in the mPFC, potentially through pathways-specific molecular changes affecting males and females differently, as suggested before in human MDD and animal models of depressive-like behaviors (Labonté et al., 2017; Seney et al., 2018; Scarpa et al., 2020; Bittar et al., 2021).

In addition to the effects mentioned above on pyramidal neurons, alterations in GABAergic interneurons have also been consistently reported in the mPFC of MDD patients. Density and size reduction of GABA interneurons, along with decreased expression of the 67kDa glutamic acid decarboxylase (GAD67) gene expression, have been reported in post-mortem mPFC tissue from MDD patients (Karolewicz et al., 2010). In contrast, more recent studies found no change in the number of SST-expressing GABAergic neurons in men and women mPFC although a reduction in SST mRNA expression levels was reported in the dorsolateral PFC and ACC with females exhibiting a much stronger decrease than men (Tripp et al., 2011; Seney et al., 2015). Here again, these findings have been supported in animal models of chronic stress. Indeed, dendritic hypertrophy was also reported in SST interneurons following chronic restraint stress in males (Gilabert-Juan et al., 2013) although, SST and neuropeptide Y protein levels were reduced in males after chronic unpredictable stress in the mPFC (Banasr et al., 2017). However, in contrast with data in humans, increases in the number of parvalbumin (PV) positive cells and PV mRNA in the PLC and ILC was reported in female mice after chronic unpredictable mild stress while in males, the number of PV positive cells was significantly increased only in the PLC with no change in PV mRNA expression (Shepard et al., 2016).

### E/I Balance Integrity in Stressed Males and Females

Overall, the sum of evidence reported here suggests that chronic stress induces a global reorganization of neuronal spine and arborization in the mPFC, disrupting the frontotemporal neuronal network’s integrity differently in males and females (Sarkar and Kabbaj, 2016; Bittar et al., 2021). Importantly, these sex-specific changes, affecting excitatory and inhibitory neuronal populations are likely to interfere with the balance between excitatory and inhibitory (E/I) tones required to maintain proper function of the mPFC (Fee et al., 2017).

At this point, whether chronic stress increases or decreases the E/I balance in the mPFC is still a matter of debate with functional studies that aim at modulating the activity of the mPFC, and report conflicting results. On one hand, studies increasing the excitability of mPFC pyramidal neurons or decreasing the inhibitory tone of GABAergic interneurons suggest that pyramidal hyperactivity in the mPFC associates with the expression of depressive-like behaviors. For instance, asynchronous optogenetic activation of pyramidal neurons in the mPFC was shown to induce the expression of depressive-like behaviors in male rats (Ferenczi et al., 2016). Previous studies showed that the optogenetic stimulation of the Stabilized Step-Function Opsin (SSFO) (Yizhar et al., 2011) in pyramidal neurons induces stable subthreshold neuronal depolarization, priming the neurons to natural endogenous stimuli (Berndt et al., 2009) and providing a more physiologically relevant increase in neuronal excitability. The activation of mPFC pyramidal neurons with this approach was shown to increase blood-oxygen-level dependent activity in the mPFC and trigger the expression of anhedonia and social avoidance with no motor or cognitive impairments in naive male rats (Ferenczi et al., 2016). Interestingly, this pattern of activity recapitulates some of the findings reported before in treatment-resistant MDD patients (Lozano et al., 2011; Li et al., 2013; Holtzheimer et al., 2017) and suggests that pyramidal neurons’ hyperactivity in the mPFC drives specific aspects of depressive-like phenotypes. This imbalance in the E/I ratio was also reported in mice following CVS. Indeed, the E/I frequency ratio was significantly shifted toward excitation in NAc-projecting mPFC neurons, with lower inhibitory inputs onto these neurons in both males and females, combined with stronger excitatory inputs, but only in stressed females (Bittar et al., 2021). In contrast, E/I imbalance in VTA-projecting mPFC neurons was only trending toward excitation with higher excitatory inputs in both sexes but no change in the inhibitory tones imposed over these neurons in both stressed males and females (Bittar et al., 2021). Interestingly, this was corroborated by pathway-specific variations in the expression of GABA and glutamate NMDA receptor subunits (Bittar et al., 2021) as a potential mechanistic explanation for these stress-induced functional changes.

Consistently, the inactivation of GABAergic inhibitory tones in the mPFC has also been shown to promote the expression of depressive-like behaviors. For instance, the acute chemogenetic inhibition of SST-expressing GABAergic interneurons in the PLC triggers anxiety-like behaviors in male and female mice (Soumier and Sibille, 2014). Chemogenetic inactivation of PV-expressing GABAergic interneurons during learned helplessness increases the escape latency and failure to escape as measures of behavioral despair (Perova et al., 2015). Furthermore, SST-KO mice exhibit depressive- and anxiety-like behaviors along with reductions of BDNF and GAD67 expression in GABA interneurons and elevated corticosterone blood levels (Lin and Sibille, 2015). By compiling human cellular, synaptic, and gene expression data from recent studies, Yao et al. (2021) generated computational models of human layer II/III local circuit. Among these models, they created a depression microcircuit model by reducing SST inhibition according to previous gene expression data from MDD patients (Seney et al., 2015). In this model, a reduction in SST inhibition leads to an increase in the pyramidal firing rate, increasing baseline activity, disrupting signal-to-noise ratio, and the increasing probabilities of both false signal detection and failed signal detection over background noise (Yao et al., 2021).

On the other hand, contrasting findings have been reported where the stimulation of pyramidal neurons in the mPFC has been associated with antidepressant responses. For instance, social withdrawal and anhedonia induced in males after 10 days of social defeat stress were shown to be reversed by optogenetic stimulations of the mPFC (Covington et al., 2010) while similar stimulation of pyramidal neurons in the infralimbic portion of the mPFC in naïve animals decreases helplessness, anhedonia, and anxiety (Fuchikami et al., 2015). Furthermore, although the acute chemogenetic inactivation of SST GABAergic interneurons in the mPFC induces anxiety-like behaviors, their prolonged inhibition over 21 days promoted antidepressant-like responses in males, increasing time spent in the open arms of the elevated plus-maze test (Soumier and Sibille, 2014).

Together, by interfering with the morphological and functional organization of pyramidal and GABAergic neurons in the mPFC, chronic stress interferes with the E/I balance controlling the function of the mPFC. The morphological adaptations observed in pyramidal and interneurons of the mPFC after chronic stress suggest that their ability to integrate and transmit correctly neuronal signals is compromised. This is leading to functional modifications that interfere with the E/I balance. A proper E/I balance assures proper responses to various stimuli received by these neurons. This being said, given the complex integrative role of the mPFC at the circuit level, it is likely that the morphological changes reported above may affect precise neuronal subpopulations that could be defined based on their inputs or outputs (Shansky et al., 2009) and by doing so, change the activity of neuronal pathways controlling emotional stress responses (Lin and Sibille, 2015; Knowland and Lim, 2018; Biselli et al., 2019; Bittar et al., 2021). In the following section, we will review the literature dissecting the functional and behavioral contributions of distinct neuronal circuits targeting or originating from the mPFC in the establishment of stress responses in males and females.

## Functional Contributions of the mPFC Circuitry in Mediating Behavioral Stress Responses

### Alterations in mPFC Afferences

The mPFC receives various afferences from cortical, subcortical and brainstem areas (Zhang et al., 2010; Gabbott et al., 2012; Linley et al., 2013; Vander Weele et al., 2018; Quessy et al., 2021) which have been shown to control several fundamental behavioral features such as memory formation, emotional processing, decision-making, attention and behavioral flexibility (Beversdorf et al., 1999; Gunaydin et al., 2014; Mitchell, 2015; Sharp, 2017; Phillips et al., 2019). With this in mind, recent studies investigating the contributions of these afferences in the elaboration and consolidation of stress responses address more specifically their roles in modulating anxiety, behavioral despair, social avoidance, and anhedonia-like behavioral features in males and females (Table 1).

**TABLE 1:** Overview of preclinical studies involving mPFC afferences in anxiety-like and depressive-like behaviors.

**Afferences**	**Species**	**Sex studied**	**Stress**	**Neuromodulatory approach**	**Effects observed**	**Tests performed**	**References**
Ventral	Mouse	Not specified	Naive	Chemogenetic activation	Anxiogenic	EPM, OFT	Parfitt et al., 2017
hippocampus	Mouse	Not specified	Naive	Chemogenetic inhibition	Anxiolytic		
(vHIPP)	Mouse	Male	Naive	Optogenetic inhibition	Anxiolytic, antidepressant	EPM, OFT, NSF	Padilla-Coreano et al., 2016
Basolateral amygdala (BLA)	Mouse	Male	Naive	Optogenetic stimulation	Anxiogenic	EPM	Lowery-Gionta et al., 2018
	Mouse	Male	Naive	Optogenetic stimulation	Anxiogenic, pro-depressive	EPM, OFT, Social exploration	Felix-Ortiz et al., 2016
	Mouse	Male	Naive	Optogenetic inhibition	Anxiolytic, antidepressant	OFT, Social exploration	
	Mouse	Both	Naive	Chemogenetic activation	Anxiogenic	EPM	Marcus et al., 2020
Locus coeruleus (LC)	Rat	Male	Naive	Chemogenetic activation	Anxiogenic, aversive	OFT, Place preference	Hirschberg et al., 2017
Mediodorsal thalamus (MDT)	Mouse	Not specified	Naive	Chemogenetic activation	Antidepressant	FST, TST	Miller et al., 2017
	Rat	Male	Naive	Optogenetic inhibition	No effect	FST	Carreno et al., 2016
	Mouse	Male	Naive	Optogenetic inhibition	No effect	EPM	Padilla-Coreano et al., 2016
Ventral tegmental area (VTA)	Mouse	Female	Naive	Optogenetic stimulation	Anxiogenic	EPM	Gunaydin et al., 2014
	Mouse	Male	Acute SDS	Optogenetic inhibition	Pro-depressive, anhedonia	Social interaction, SPT	Chaudhury et al., 2013
	Mouse	Male	Acute SDS	Optogenetic stimulation	No effect		
	Mouse	Male	CSDS	Optogenetic stimulation	Antidepressant	Social interaction	Friedman et al., 2014

Neurons from the VTA projecting to the mPFC constitute the mesocortical pathway. The VTA itself, and more importantly, via its projections to the mPFC has been extensively studied for its contribution to aversive stimuli processing (Gunaydin et al., 2014; Lammel et al., 2014). Functional studies have also revealed the crucial impact of this dopaminergic pathway on the regulation of anxiety and social behaviors involved in emotional stress responses (Friedman et al., 2014; Liu D. et al., 2020). For instance, the optogenetic stimulation of the VTA-mPFC pathway in stress naïve female mice had an anxiogenic effect and triggered aversion in a conditioned place preference test (Gunaydin et al., 2014). Interestingly, this was not associated with any change in the social interaction test, suggesting that the mesocortical pathway is regulating aversion but not sociability. Additionally, the acute stimulation of this pathway had no noticeable effect on sociability and anhedonic behaviors in males after sub-threshold social defeat stress while the optogenetic inhibition of the VTA-mPFC pathway following sub-threshold CSDS was shown to induce social withdrawal in males (Chaudhury et al., 2013). Interestingly, the VTA-mPFC firing rate was significantly reduced in susceptible animals but not in resilient and control, suggesting that decreased activity of this pathway causes social avoidance in susceptible males (Chaudhury et al., 2013). Moreover, the density of dopamine axon terminals in the layers V and VI of the mPFC is reduced in male and female mice after CSDS (Quessy et al., 2021). Additionally, repeated optogenetic stimulation of mPFC-projecting VTA neurons in mice that underwent CSDS reversed social avoidance (Friedman et al., 2014). Another study also reported an increased c-Fos expression in the mPFC after optogenetic stimulation of the VTA dopamine neurons (Gunaydin et al., 2014).

The ventral hippocampus (vHIPP) sends dense glutamatergic projections to the mPFC with denser inputs in the vmPFC (Goldman-Rakic et al., 1984). This pathway was previously shown to be particularly relevant to social memory, emotional and motivational behaviors (Bannerman et al., 2004; Sigurdsson and Duvarci, 2016; Phillips et al., 2019). Interestingly, functional studies revealed the bidirectional control of this projection over the expression of anxiety-like behaviors (Padilla-Coreano et al., 2016; Parfitt et al., 2017). For instance, acute chemogenetic stimulation of mPFC-projecting vHIPP neurons in stress naïve mice induced anxiety-like behaviors with a decreased time spent in the open arms of the elevated plus-maze, an increased latency to feed in the novelty-suppressed feeding test, and a decreased time in the center in the open field test (Parfitt et al., 2017). On the contrary, inhibition of this pathway, either with chemogenetic or optogenetic approaches, alleviates anxiety-like behaviors in males as shown by the increased time spent in the open arms of the elevated plus-maze test (Padilla-Coreano et al., 2016). More work will be required to investigate whether this circuit contributes to the same behavior in females.

Projections from the basolateral amygdala (BLA) to the mPFC are also directly involved in the control of anxiety-like behaviors in mice. The mPFC receives excitatory glutamatergic inputs from the BLA targeting dmPFC and vmPFC in layers II/III and V (Gabbott et al., 2012). Traditionally, the BLA has been associated with the control of fear, anxiety, and reward learning (Wassum and Izquierdo, 2015; Sharp, 2017) and its projections to the mPFC have been implicated in anxiety-related behaviors (Liu et al., 2015; Liu W.-Z. et al., 2020; Felix-Ortiz et al., 2016). For instance, lesioning the BLA in stress naïve rats induced a reduction of spine density on the basal dendritic tree of pyramidal neurons without dendritic attrition in the mPFC (Liu et al., 2015). Furthermore, the optogenetic activation of this pathway in stress naïve male mice increased anxiety (Felix-Ortiz et al., 2016; Lowery-Gionta et al., 2018), and social avoidance (Felix-Ortiz et al., 2016) while the optogenetic inhibition of these pathways induced opposite behavioral effects (Felix-Ortiz et al., 2016). Moreover, a recent study highlighted the anxiogenic effect of the BLA-mPFC pathway in stress-naive males and females with acute chemogenetic stimulation, decreasing the time spent in the open arms during the elevated plus-maze test (Marcus et al., 2020). The anxiogenic effect of the BLA-mPFC pathway was exacerbated by the viral deletion of cannabinoid receptor CB1 in the mPFC-projecting BLA neurons, suggesting the role of endocannabinoid negative feedback in anxiety regulation in the BLA-mPFC circuit. This manipulation impairs the electrophysiological regulation of mPFC neurons receiving BLA inputs (Marcus et al., 2020).

The LC sends dense noradrenergic projections distributed across the mPFC superficial layers I and II/III (Nestler et al., 2008). The LC has been involved in arousal, attention, cognitive flexibility, decision making, and emotions (Beversdorf et al., 1999; Nestler et al., 2008; Benarroch, 2009) and more recently, its projections to the mPFC have been involved in the control of anxiety-like behaviors in mice (Hirschberg et al., 2017). Indeed, chemogenetic activation of the LC to mPFC neuronal pathway was shown to trigger anxiety-like behaviors in the open field test and aversion in the place preference test in naïve male rats (Hirschberg et al., 2017). Similarly, chemogenetic activation of this pathway was shown to improve set-shifting behaviors in conditioning chamber tests (Cope et al., 2019), highlighting the importance of this pathway in adaptive behaviors during learning and its relevance to stress-copying strategies.

Finally, the mediodorsal thalamus (MDT) integrates inputs from various brain regions such as the amygdala, VTA, and DRN and transmits this information to the mPFC (Aggleton and Mishkin, 1984; Groenewegen, 1988; García-Cabezas et al., 2009). The MDT has been shown to control the integration of emotional information from these limbic structures and is involved in learning, decision-making process (Mitchell, 2015), and behavioral flexibility (Floresco et al., 1999; Parnaudeau et al., 2015). MDT-mPFC connectivity has been shown to be impaired in treatment-resistant MDD patients (Li et al., 2013). Previous studies showed that the chemogenetic activation of the MDT to mPFC neuronal pathway decreases behavioral despair with reduced immobility in the tail suspension test and forced swim test in naive mice (Miller et al., 2017). On the contrary, optogenetic inhibition of MDT-mPFC neurons was shown to have no effect on depressive-like behaviors in the forced swim test and elevated plus maze in males (Carreno et al., 2016; Padilla-Coreano et al., 2016). Interestingly, the mPFC neurons receiving MDT inputs express GluN2B receptor subunit, accounting for approximately 35% of NMDAR-mediated currents (Miller et al., 2017). Viral deletion of GluN2B significantly enhanced MDT-mPFC but not vHIPP-mPFC EPSC amplitude in the mPFC layer III pyramidal neurons (Miller et al., 2017). GluN2B receptors are critical for ketamine’s antidepressant effect (Miller et al., 2014) supporting a mechanism of action for ketamine to engage preferentially MDT-mPFC inputs rather than the vHIPP-mPFC pathway.

### Alterations in mPFC Outputs

As detailed above, the mPFC projects to several brain regions involved in the control of emotions and reward processing (Gunaydin et al., 2014; Floresco, 2015; Wassum and Izquierdo, 2015). As for the contribution of its afferences, functional studies have also dissected the roles of specific neuronal pathways in the expression of stress susceptibility and resilience (Table 2).

**TABLE 2:** Overview of preclinical studies involving mPFC efferences in anxiety-like and depressive-like behaviors.

**Efferences**	**Species**	**Sex studied**	**Stress**	**Neuromodulatory approach**	**Effects observed**	**Tests performed**	**References**
Basomedial amygdala (BMA)	Mouse	Both	Naive	Optogenetic stimulation	Anxiolytic	OFT, EPM	Adhikari et al., 2015
	Mouse	Both	Naive	Optogenetic inhibition	Anxiogenic		
Basolateral amygdala (BLA)	Mouse	Male	Acute SDS	Optogenetic stimulation	Anxiolytic	EPM	Vialou et al., 2014
	Mouse	Male	Chronic restraint stress	Optogenetic stimulation	Anxiolytic	OFT, EPM	Liu W.-Z. et al., 2020
Amygdala	Mouse	Male	CSDS	Chemogenetic activation	Antidepressant	Social interaction	Hultman et al., 2016
Nucleus accumbens (NAc)	Mouse	Male	CSDS	Optogenetic stimulation	Antidepressant	Social interaction	Bagot et al., 2015
	Mouse	Male	Acute SDS	Optogenetic stimulation	Pro-depressive, anhedonia	Social interaction, SPT	Vialou et al., 2014
	Mouse	Both	Naive	Optogenetic stimulation	Pro-depressive	Linear 3-chamber assay	Murugan et al., 2017
	Mouse	Both	sCVS	Chemogenetic activation	Anxogenic, pro-depressive	NSF, EPM, FST, Splash test	Bittar et al., 2021
	Mouse	Both	sCVS	Chemogenetic inhibition	Anxiolytic, antidepressant		
Dorsal raphe nucleus (DRN)	Mouse	Male	CSDS	Optogenetic inhibition	Pro-depressive	Social interaction	Challis et al., 2014
	Mouse	Male	CSDS	Optogenetic stimulation	Antidepressant		
	Rat	Female	Inescapable tailshock	Optogenetic inhibition	Pro-depressive	Social interaction	Dolzani et al., 2018
	Rat	Male	Naive	Optogenetic stimulation	Antidepressant	FST	Warden et al., 2012

Amongst the distinct neuronal circuits emerging from the mPFC, the corticoaccumbal pathway has received considerable interest regarding its involvement in the expression of stress susceptibility or resilience. For instance, the optogenetic acute stimulation of pyramidal mPFC neurons projecting to the NAc was shown to promote anxiety in the open field test in naïve males but did not change the immobility in the forced swim test or the interaction time in the social interaction test (Bagot et al., 2015). On the contrary, acute stimulation of the same neurons after CSDS did not trigger any change in anxiety and behavioral despair in the open field and the forced swim tests. However, it promoted resilience to social stress by increasing social interaction after CSDS in males (Bagot et al., 2015). Elevated activity in the NAc-projecting mPFC neurons was also reported during pro-hedonic reward-seeking in mice (Otis et al., 2017). On the contrary, stimulation of the same pathway after acute social defeat increased social avoidance and elicited anhedonia in males (Vialou et al., 2014). Consistently, continuous optogenetic stimulation of mPFC nerve terminals in the NAc reduced social interaction (Murugan et al., 2017). Interestingly, the acute chemogenetic activation of the corticoaccumbal pathway was shown to induce no behavioral impact in both stress naive males or females (Bittar et al., 2021). However, when combined with subthreshold CVS, the same stimulation paradigm was sufficient to induce anxiety and behavioral despair in both sexes (Bittar et al., 2021). Interestingly, inhibition of the same population after CVS was shown to rescue anxiety and despair phenotypes in females but not in males, suggesting that the corticoaccumbal pathway may be necessary to induce anxiety and despair in females but not in males (Bittar et al., 2021). Therefore, these contrasting results suggest that activity patterns of the cortico-accumbal pathway, which can be modulated differently by acute and chronic stress (Vialou et al., 2014; Bagot et al., 2015; Bittar et al., 2021), is particularly relevant in the determination of the proper behavioral response to stress, especially regarding anxious, hedonic, and social behaviors.

The contribution of the mPFC projections to the amygdala, and more specifically to its different nuclei, has also been extensively studied in the context of stress responses. Indeed, optogenetic stimulation of the vmPFC neurons projecting to the basomedial nucleus of the amygdala (BMA) was shown to decrease anxiety-like behaviors with increased time spent in the open arms of the elevated plus-maze in both male and female mice chronically exposed to corticosterone (Adhikari et al., 2015). On the contrary, the inhibition of this pathway decreased the time spent in open arms in both sexes (Adhikari et al., 2015). In comparison, optogenetic stimulation of the mPFC neurons projecting to the BLA decreased anxiety behaviors in the elevated plus-maze but had no effect on social avoidance and anhedonia after acute social defeat (Vialou et al., 2014) and chronic restraint stress (Liu W.-Z. et al., 2020) in male mice. Finally, chemogenetic activation of mPFC afferences to the amygdala during social interaction test reduced CSDS-induced social avoidance in susceptible male mice (Hultman et al., 2016).

Additionally, mPFC neurons projecting to the dorsal raphe nucleus (DRN) were shown to control specific aspects of social behaviors. Optogenetic inhibition of this pathway decreases social avoidance after CSDS in male mice (Challis et al., 2014). Another study using chemogenetic approaches showed that the inhibition of this pathway in female rats exposed to inescapable tail shocks increases exploration in the juvenile social exploration test (Dolzani et al., 2018). Similarly, the optogenetic stimulation of mPFC-DRN neurons in naïve male rats decreased helplessness and behavioral despair by increasing the kicking and decreasing immobility in the forced swim test (Warden et al., 2012). Interestingly, high-frequency deep brain stimulation of the mPFC ILC in naive male rats triggered a decreased firing rate in the DRN (Srejic et al., 2015). This decrease is due to the activation of GABAergic interneurons in the DRN, inhibiting the principal neurons. Altogether, these studies suggest that mPFC neurons projecting to the DRN target the GABA interneurons and trigger their activation, leading to a decrease in DRN principal neuron activity (Warden et al., 2012; Challis et al., 2014; Srejic et al., 2015).

**FIGURE 3:**
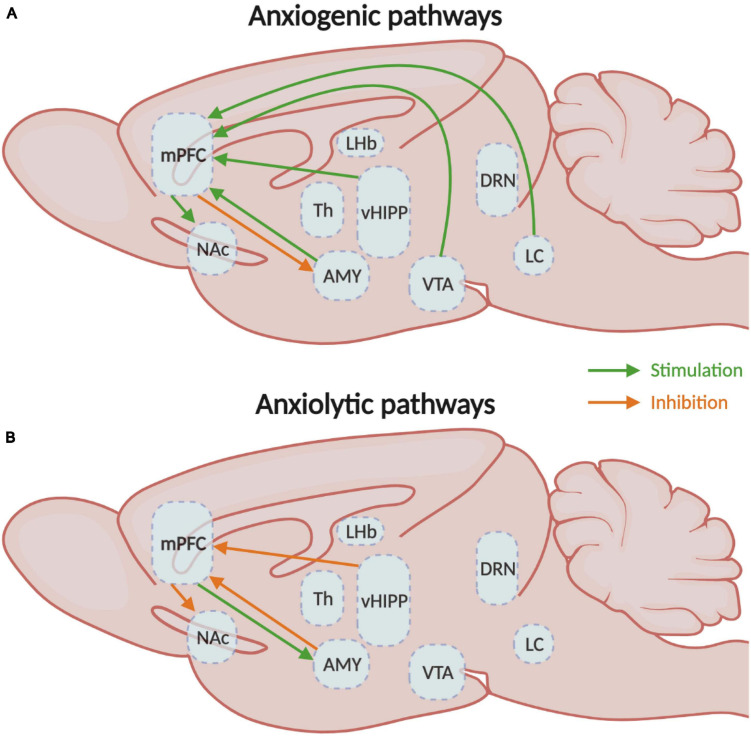
Medial prefrontal circuitry involved in anxiety-like behaviors. **(A)** Schematic representation of the mPFC circuits involved in the expression of either **(A)** anxiogenic or **(B)** anxiolytic behavioral responses. mPFC, medial prefrontal cortex; NAc, nucleus accumbens; Th, thalamus; vHIPP, ventral hippocampus; VTA, ventral tegmental area.

**FIGURE 4:**
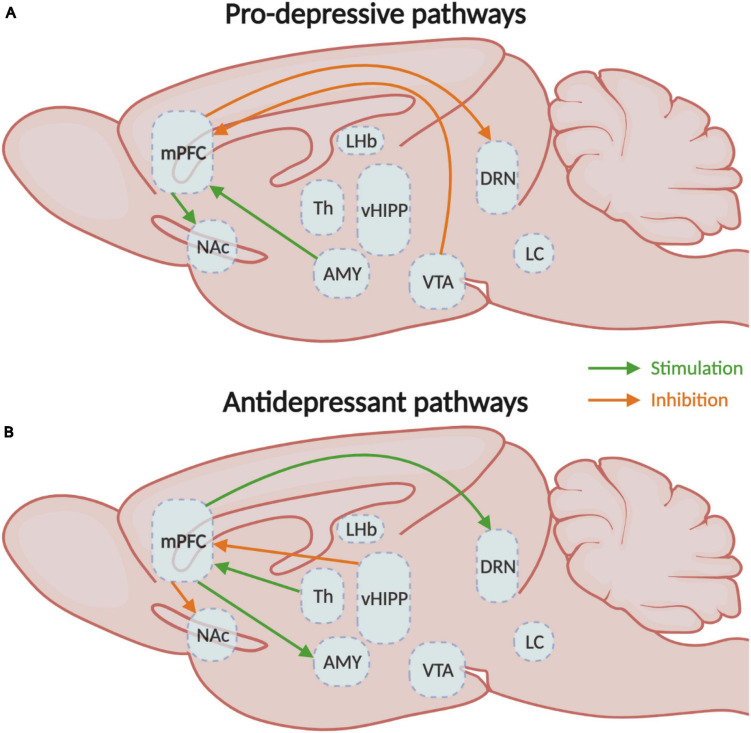
Medial prefrontal circuitry involved in depressive-like behaviors. **(A)** Schematic representation of the mPFC circuits involved in the expression of either **(A)** depressive- or **(B)** antidepressant-like behavioral responses. mPFC, medial prefrontal cortex; NAc, nucleus accumbens; Th, thalamus; vHIPP, ventral hippocampus; VTA, ventral tegmental area. CSDS, chronic social defeat stress; EPM, elevated plus-maze test; FST, forced swim test; NSF, novelty-suppressed feeding test; OFT, open field test; sCVS, subthreshold chronic variable stress; SDS, social defeat stress; SPT, sucrose preference test.

In consequence, the activation of the mPFC neurons that project to the LC, vHIPP, BLA, and VTA elicits anxiety, while the inhibition of vHIPP-mPFC and BLA-mPFC have anxiolytic effects in rodents (Figure 3). Activation of the BLA-mPFC neurons also has a pro-depressive impact, as well as inhibiting the VTA-mPFC pathway (Figure 4). Globally, the functional changes reported here argue in favor of the increased activity in the mPFC associated with anxiety and depressive-like behaviors. These findings also inform us on the probable direction of the E/I imbalance after stress exposure, the balance shifting toward excitation in this case. While we have a better understanding of these pathways’ contributions in males, studies investigating their contributions in females are still lacking. Despite this limitation, the little evidence we have from studies comparing males and females suggests that specific pathways such as the corticoaccumbal pathway may contribute differently to the expression of emotional stress responses in both sexes.

## Conclusion and Future Perspectives

The sum of evidence described in this review strongly supports the role of the mPFC as a hub region in the integration and consolidation of emotional responses to chronic stress in males and females (Murrough et al., 2011; Soumier and Sibille, 2014; Ferenczi et al., 2016). This complex process appears to involve a series of morphological and anatomical alterations (Radley et al., 2004; Gilabert-Juan et al., 2013; Sarkar and Kabbaj, 2016; Bittar et al., 2021) combined with changes in the strength of mPFC inputs from several brain structures (Marcus et al., 2020; Bittar et al., 2021). Ultimately, these modifications induced by stress interfere with the E/I balance in the mPFC in a sex-specific fashion and finally with the top-down processing of sensory and emotional information. As of now, several studies investigated the contribution of neuronal pathways projecting to the mPFC (Gunaydin et al., 2014; Parfitt et al., 2017; Cope et al., 2019; Marcus et al., 2020) and, while others investigated the contribution of this structure in mediating stress responses (Challis et al., 2014; Vialou et al., 2014; Adhikari et al., 2015; Bittar et al., 2021), more work is required to dissect the contribution of distinct neuronal populations within the mPFC projecting to other brain structures. Indeed, with the development of more sophisticated functional and behavioral approaches, it is becoming increasingly clear that distinct neuronal pathways emerging from the mPFC control specific behavioral features within complex stress responses differently in males and females. Additionally, it will be important in future studies to consider the contribution of the different sub-structures of the PFC such as the ACC, the PLC and ILC, and the orbitofrontal cortex in the expression of complex stress responses in males and females.

Now, another crucial aspect to consider in future studies is the type of stress used to induce anxiety- and depressive-like behaviors in animals, which based on the literature reviewed above, appear to impose distinct changes on the activity of mPFC neurons and circuits. Indeed, stress paradigms involving mainly physical stressors are likely to impact the mPFC circuitry differently than those involving psychosocial stressors (Nestler and Hyman, 2010). Such distinctions have been shown to be of particular importance in recent studies of stress-induced transcriptional alterations in the brain from different mouse models of depressive-like behaviors (Labonté et al., 2017; Scarpa et al., 2020). Understanding how these different types of stress impact the activity of neuronal circuits in males and females to ultimately induce the expression of a complex stress response will be of the upmost importance to identify the cellular substrate to target with therapeutic interventions. Indeed, by combining molecular and pharmacological approaches with the strategies elaborated here, one will start to delineate the foundations for new therapeutic interventions that target the neuronal substrate responsible for precise behavioral manifestations rather than a complex and heterogenous stress phenotype.

## Author Contributions

TPB and BL contributed to the literature review and the redaction of the manuscript. Both authors contributed to the article and approved the submitted version.

## Conflict of Interest

The authors declare that the research was conducted in the absence of any commercial or financial relationships that could be construed as a potential conflict of interest.
